# Supplementary material for: Are carbon emissions trading and green financial instruments synergistic? -Comprehensive quantitative research based on content analysis
Source: PLoS One. 2024 Mar 7;19(3):e0298601. doi: 10.1371/journal.pone.0298601 (PMC10919601; doi:10.1371/journal.pone.0298601)
Supplement: S1 Data — (ZIP) [file pone.0298601.s001.zip › Data/地级市Z_F工作报告.zip!/░▓╗╒/░÷▓║╩╨/└┤╫╘╣1⁄2╓┌║┼í╛┬φ┐╦╩2╛▌═°í┐_2014.docx]

各位代表： 
    现在，我代表市人民政府向大会报告工作，请予审议，并请市政协委员和其他列席人员提出意见。 
    一、攻坚克难，经济社会发展实现新突破 
    刚刚过去的2013年，面对严峻复杂的宏观形势和经济下行的较大压力，全市上下在重返全省第一方阵宏伟目标的感召、指引下，在市委的坚强领导下，认真贯彻党的十八大和十八届三中全会精神，大力弘扬先进思想文化，切实加强和改进作风建设，全力以赴稳增长、调结构、促改革、惠民生，全力落实十五届人大一次会议确定的目标任务，主要经济指标增速继续好于全省、领先皖北，部分指标总量进位、突破历史，社会大局和谐稳定，实现了本届政府良好开局。预计，全市地区生产总值有望迈上千亿元新台阶，同比增长11.3%，增速居全省前列。一、二、三产增加值分别增长3%、14.5%、10%。固定资产投资突破千亿元，增长21.5%。在减免抵退各类税费30多亿元、着力压缩非税收入的同时，实现财政收入182.8亿元，跃居全省第5，增长11%。其中地方收入92.8亿元，增长18.4%。城镇居民人均可支配收入22733元，增长10.2%。农民人均纯收入8750元，增长14%。社会消费品零售总额424.6亿元，增长14.3%。居民消费价格涨幅低于全省平均水平，万元地区生产总值综合能耗、主要污染物减排持续下降。 
    (一)全力攻坚产业发展，经济实力进一步提升。 
    工业经济逆势上扬。开展服务企业创建百佳行动，兑现各类促进工业经济发展资金25亿元。新增规模以上工业企业112户，实现规模以上工业增加值480亿元，增长15.3%。中建材电子信息显示超薄基板、德豪润达LED产业化基地一期等74个项目建成投产，首文碳纤维生产线达到量产水平，中科电力装备产业园、台玻电子玻璃纤维等85个项目开工建设。新型显示产业进入国家战略性新兴产业集聚发展试点。高新区成功创建国家级(硅基)新材料新型工业化产业示范基地，经开区长淮卫临港产业园前期工作扎实推进，蚌埠铜陵现代产业园一期基础设施建设全面完成。全市工业化率达45%。 
    三农工作提质增效。深入实施农业百佳工程，粮食总产达263.6万吨，实现十连增。蚌埠国家农业科技园区获批设立，首家农业院士工作站、国家级生物有机肥创制重点实验室落户蚌埠。新增省级农业产业化龙头企业14家、农民专业合作社560个、家庭农场152个。增加土地流转40万亩，土地流转率达40%，高于全省11个百分点。新中国治淮以来投资最大单项水利工程——— 淮干蚌浮段行蓄洪区调整和建设工程顺利开工。建设高标准基本农田46.66万亩，新增有效灌溉面积8.7万亩。首批129个美好乡村中心村建设全面启动，46个重点示范村效果初现。 
    第三产业集聚升级。全国家政服务体系、中小商贸流通企业公共服务平台等试点扎实推进，省级商贸示范区启动建设。义乌国际商贸城、国际汽车城、万达一期等重点商贸项目建成运营，新增限额以上商贸企业150户。小商品批发市场划行归市试点顺利推进。蚌埠(皖北)保税物流中心申建工作有序开展，蚌埠铁路无水港开工建设。举办首届蚌埠花博会，湖上升明月古民居博览园、大明文化产业园等项目加快推进，南山豪生国际大酒店建成开业。星宇文化创意产业园一期开园，盛大游戏、华立科技等知名动漫游戏企业入驻园区。 
    (二)大力推进城市建设，综合环境进一步改善。 
    承载能力不断提升。《蚌埠市城市总体规划(2012-2030)》编制完成，并通过省规委会审议。部分区域行政区划调整稳步推进。实施城市大建设项目160个，完成投资超200亿元。市区征迁617万平方米、拆违100万平方米，交付净地2.82万亩。蚌固一级公路建成通车，国道104五河段、省道307怀远段一级公路改建开工。长淮卫淮河大桥、黄山大道贯通、胜利东路改造等工程开工建设，东海大道、淮上大道改扩建和解放路、朝阳路提升改造工程顺利完成。 
    人居环境持续优化。回迁安置棚改居民16500户，棚改蚌埠模式得到国务院和省主要领导肯定，在全国、全省推广。开工建设保障性安居工程33630套，廉租房实物配租9099户。102户市区以船为家渔民上岸安居。开展千家万户百日节能自愿行动，实行全年全市全面秸秆禁烧。天河应急水源改建竣工，城南污水处理厂一期建成运营。实施千万亩森林增长工程，植树造林13.1万亩，是“十一五”造林总和的2倍多。开展绿满珠城行动，新增森林长廊86公里，新增、改造绿地179万平方米。张公山公园、龙子湖北公园全新开放，陶山植物园开工建设。龙子湖及周边综合治理和生态开发项目一期工程获鲁班奖。 
    市容市貌明显改观。城市南、北出入口综合环境大幅提升。投入1600万元新购环卫机械设备38台，推进保洁属地化、机械化和市场化。实施老旧小区环境提升工程，启动“三线三边”环境治理。大力开展“三车”整治，平稳取缔人力三轮车。强化城市管理，占道经营、乱搭乱建等现象得到有效遏制。 
    (三)着力扩大有效投入，发展后劲进一步增强。 
    项目投资成效显著。完成“3461”项目投资630亿元，全市在建亿元以上项目352个。晟光科技电容式触摸屏二期、方兴科技减薄玻璃一期、依爱电子产业园一期、大富机电射频器件二期等138个项目建成投产，北方通用MEMS产业基地、百特纳米新材料等281个项目加速实施，固镇亿只肉鸡产业化、贵联大型印刷包装生产基地等158个项目开工建设。新增谋划项目167个，储备规模超4000亿元。争取中央、省补助资金7亿元。 
    招商引资持续升温。积极参与皖港经贸交流、安徽省与全国知名民营企业家合作发展等活动，百家民企进皖北蚌埠行取得丰硕成果，民企合作项目数、开工率全省第1。成功举办佛山、汕头恳谈会等招商活动，积极承办第十届全国动漫游戏行业年会，大力开展专题招商、产业链招商、以商招商，台湾巨力旺食品观光产业园、新大地汽车产业园、台一国际等18个10亿元以上项目落户蚌埠。引进市外亿元以上项目资金550亿元、增长31%，实际利用外商直接投资9.5亿美元、增长30%。 
    融资保障积极有效。深入开展金融服务拓展年、金融服务进百企活动，新增贷款180亿元，存贷款增速分别居全省第1和第2。新型城镇化试点获国开行50亿元授信，城投公司发行企业债券16亿元。佳先股份、昌盛电子通过新三板挂牌审核。商标权、专利权质押贷款总额突破5亿元。怀远农合行和五河、固镇农信社改制农商行获批。 
    (四)聚力深化改革创新，发展活力进一步释放。 
    科技创新步伐加快。蝉联全国科技进步先进市，荣获全省唯一国家科学技术进步奖。新型高分子材料产业集群列入全国创新型产业集群试点(培育)。新认定高新技术企业38户、省级创新型企业7户，实现高新技术产业产值580亿元，增长23%。新增省级以上研发平台15个，51家企业新列入企业股权和分红激励试点范围，12家企业完成试点方案备案审批。发明专利授权量391件，增长31%。 
机制创新力度加大。修订政府工作规则，建立市政府负责同志联系县区、协同招商等制度。开展市级行政审批项目集中清理，精简下放审批项目435项。完善政府购买公共服务、标后监管制度，市公共资源交易中心纳入国家级服务标准化建设试点。调整山林管辖权，制定河湖管理办法，建立效能建设与机关编制管理挂钩机制。稳步推进大科制改革试点。启用一体化电子政务综合应用系统，建立市长热线服务中心。启动集团化办学、办医，组建教育集团5家、医疗集团2家。市直机关一般性支出压缩5%以上，全市三公经费下降13.8%。 
    改革开放纵深推进。率先出台促进民营经济发展政策，新增私营企业3101户、个体工商户19485户，17家企业入围省民企百强。成功转让3家污水处理厂特许经营权，城市污水处理率达98.5%。北泰汽配园破产清算和重整取得阶段性成果。列入铁路货运改革试点市，开通蚌沪铁海联运定时定点快线，进出口货运量超国家一类口岸标准。实现进出口总额15.8亿美元，增长28.7%。 
    (五)致力保障改善民生，群众福祉进一步增加。 
    民生工程深入实施。投入资金43.5亿元。全面完成公共文化场馆开放、生活无着人员社会救助等33项省民生工程。12项市级民生工程圆满完成，投放新能源公交车211辆，新建、改造标准化农贸市场6个、路灯10080盏、公厕20所，创建示范社区8个，为2085个班级配备“班班通”多媒体远程教学设备，发放城乡老人高龄津贴2828万元，资助贫困大学生和困难家庭学龄前儿童417万元，补助“一户多残”家庭296万元。全市民生支出占财政总支出的83%。 
    社会事业协调发展。新增城镇就业人员6.6万人，创历史新高。最低工资、失业保险金、企业退休人员养老金等标准全面提高，发放城乡低保救助金2.9亿元，逐步兑现企业退休人员计划生育补助。新建、扩建幼儿园129个，北师大蚌埠附属学校开工建设，二中新校区投入使用。市一院晋升三级综合医院，上海长征医院合作医院正式签约。群众体育工作获评全国先进。成功举办禹会村遗址与淮河流域文明研讨会，学术界认定了蚌埠是禹会诸侯之地、禹会村遗址是国家起源重要的学术支撑、淮河流域是中华文明的重要发源地之一。花鼓灯作品《大河湾的孩子们》获国际金奖。板车女孩黄凤入选全国“最美孝心少年”。新博物馆、档案馆、规划馆主体工程和市民广场基本建成，市图书馆晋升国家一级馆。第二轮修志任务全面完成。民族宗教工作跨入全省先进行列。外事、侨务、港澳台工作取得新成绩。男女平等发展和儿童优先原则有效落实。首届青年创业大赛成功举办。敬老文明号创建位居全省第2。残疾人生存和社会环境明显改善。国家特种玻璃质量监督检验中心建成投用。统计工作基础和水平明显提高。新增住房公积金缴存人数10348人。应急管理体系逐步完善。社会防震减灾意识显著提升。人防工作获南京军区通报表彰。实现全省双拥模范城七连冠。蚌埠舰正式入列，舰上官兵被授予蚌埠荣誉市民。驻蚌部队和民兵预备役人员在地方经济社会发展中作出重要贡献。 
    社会管理切实加强。做好信访、法律援助、人民调解等工作，社会矛盾有效化解。深入开展平安蚌埠建设，加大消防装备投入，注重安全生产，群众安全感增强。推进依法行政，强化监察和审计工作，严肃查处一批违纪违法案件。 
    一年来，我们认真办理市人大代表议案、建议147件，市政协提案549件。市人大常委会围绕经济社会发展重要工作依法行使监督权、决定权，就事关发展的重大问题及时作出决议决定，听取审议政府有关工作报告，提出审议意见；市政协积极组织促进民营经济发展专题协商，开展文化创意产业发展、新型城镇化建设等调研，有力促进了政府相关工作的落实，推动了经济社会持续健康较快发展。 
    回首2013年，谋划凝聚心血，落实洒满汗水，成绩来之不易。这是省委、省政府高度重视、关心关怀的结果，是市委正确领导、科学决策、周密部署、全面协调的结果，是市人大、市政协有效监督、鼎力支持的结果，是全市人民团结实干、拼搏奉献的结果。在此，我谨代表市人民政府，向全市人民，向人大代表、政协委员，向离退休老同志，向各民主党派、工商联、无党派人士，向各人民团体，向驻蚌解放军、武警官兵和政法干警，向国家和省驻蚌单位，向所有关心和支持蚌埠发展的各界朋友，表示衷心的感谢和崇高的敬意！ 
    各位代表，在充分肯定成绩的同时，我们清醒地认识到，在应对严峻复杂宏观形势和推进任务落实方面，自身还有督导不力、落实不够之处。虽然市人大常委会实事求是地依法调整了年度计划指标，但我们在感谢的同时，深感压力巨大，深思自身不足，深知更需努力。我们也清醒地认识到，蚌埠仍处在边打基础边增强集聚辐射能力的转型时期，持续赶超、不断跨越，重返全省第一方阵的潜力还需进一步挖掘，内生动力还需进一步提振和培育。存在的问题主要是：综合实力还不够强，中心城市的功能带动力和辐射影响力不足，牵动性强的大项目、好项目不多；产业竞争力还不够强，缺乏整机、整器、整车类终端产品，龙头企业偏少，重点产业聚集度不高；城乡发展承载力还不够强，基础设施仍不完善，城市老旧小区提升改造任务繁重，城乡还存在环境脏乱差现象；社会民生服务能力还不够强，教育、医疗、交通、安全等公共服务与百姓和客商需求存在差距，就业和社会保障压力较大，改善民生和社会治理任务艰巨；改革创新动力还不够强，守旧思想依然存在，制约加快发展的体制机制障碍仍然较多，政府职能转变速度还不能适应发展新要求；文明创建和城乡管理的能力还不够强，部分市民热爱蚌埠、发展蚌埠、奉献蚌埠的激情还需进一步引导和释放，等等。这些都是我们继续努力突破的难点，也是今后工作的重点。 
    二、抢抓机遇，持续赶超再跨越 
    2014年，是必须紧紧抓住而且应该大有作为的机遇之年。党的十八届三中全会拉开了新一轮改革开放的大幕，必将为打造蚌埠经济升级版、重返全省第一方阵提供强大持久动力。国内经济逐步企稳回暖、稳中向好，省委、省政府加快皖北振兴、支持区域性中心城市建设等力度进一步加大，中原经济区、合芜蚌自主创新综合试验区、振兴老工业基地等政策叠加效应将加速释放，蚌埠仍将处于加速发展的黄金机遇期。同时，千亿GDP的经济总量将蚌埠带入了一个崭新的发展高度，220平方公里的建成区用地规模以及区划调整实施、西部城区整体提升改造、方邱湖行蓄洪区调整为蚌埠打开了广阔的发展空间。为此，我们要牢固树立时不我待的机遇意识，坚持稳中求进、在进字上下功夫，坚持改革创新、在变字上求突破，担负起加快速度、做大总量和转型升级、提高质量的双重责任，切实把改革释放的红利转化为实实在在的发展成果，继续保持加速崛起的强劲势头。 
    2014年政府工作的总体要求是：全面贯彻党的十八大和十八届三中全会精神，以邓小平理论、“三个代表”重要思想、科学发展观为指导，突出五位一体，坚持四化同步，紧扣重返全省第一方阵的目标，稳中求进，改革创新，聚焦大产业、大招商、大项目、大平台、大民生，推动总量、速度和质量、效益双提升，进一步增强全市经济社会可持续跨越发展能力。 
    2014年全市经济社会发展的主要预期目标是：地区生产总值增长11%以上，一、二、三产分别增长3.5%、14.5%和10.5%，规模以上工业增加值增长15.5%；财政收入增长11%以上；固定资产投资增长16%以上；社会消费品零售总额增长13%；进出口总额增长15%；城乡居民人均可支配收入与地区生产总值同步增长；城镇登记失业率、人口出生率、居民消费价格涨幅、单位生产总值能耗、国家要求削减的主要污染物排放均控制在省下达的目标以内。主要经济指标增速确保高于全省、位居前列。 
实现上述目标任务，我们将重点抓好以下九方面工作： 
    (一)突出工业强市，力促工业升级提速。强化工业主导作用，把工业强市作为重返全省第一方阵的核心战略，聚集资源要素，聚焦龙头企业，聚力终端产品，着力推动企业提质、园区提速、产业提升，加速新型工业化进程，工业总产值突破2000亿元。 
    推动产业升级。以“5116”重点产业发展工程为抓手，以战略性新兴产业为突破，以工业发展扶持政策为保障，加快工业转型升级。坚持上下联动，竣工投产北方通用MEMS产业基地一期、方兴科技电容式触摸屏等项目，开工建设德豪润达倒装芯片、今上OGS导电膜等项目，延伸电子信息产业链条。坚持龙头带动，加快建设台玻电子玻璃纤维一期、配天工业智能机器人研发及产业化、中科电力装备产业园等项目，推进兆德TC生物技术暨生物新药、新大地汽车产业园等重大项目尽快实施，建成意义环保退市进园一期、海华医药中间体、凯特雅新型塑木型材一期等项目，提升高端装备、生物医药、新材料等先进制造业水平。以高新技术和先进适用技术改造提升食品、纺织、建材等传统产业，加快现代牧业一体化、蚌埠卷烟厂新址提升、华芳纺织三期等项目建设。大力推进丰原集团、中粮生化等重点化工企业异地技改、整体搬迁，启动西部城区整体提升改造，让老企业焕发新生机、老城区脱胎新面貌。 
    壮大企业规模。扎实开展服务企业拓展年活动，大力扶持实体经济，新增规模以上工业企业120户、产值10亿元以上企业5户。深化国有企业改革，推动国有企业引进战略投资者和企业间整合重组，积极发展混合所有制经济。全面深化与央企战略合作，推进玻璃设计院8.5代TFT液晶显示基板和金刚玻璃研发生产、中电科41所仪器仪表产业园、北方通用微光摄像仪、中粮生物能源产业园等重大项目尽快落地。加快推进中小企业产业园建设，建成中小企业标准化厂房70万平方米。实施中小企业培育雏鹰工程，新增专精特新中小企业50户，培育更多增长源。 
    做强园区经济。出台开发区特色发展实施意见，引导开发区错位发展、集聚发展。加快高新区、马城工业园融合发展，力争经济开发区晋升国家级。推动蚌埠工业园、沫河口工业园联动升级，支持怀远县、五河县新扩建省级开发区，全面启动蚌埠铜陵现代产业园二期基础设施建设。 
    (二)巩固农业基础，大幅提升现代化水平。 
    坚持四化同步，深入实施农业百佳工程，大力提升农业科技水平和产业化程度，延伸农业、增值农业，稳定农业生产，稳定农产品供应，稳定二三产业资源要素供给，提高物资技术和装备水平，加快农业大市向农业强市转变。 
    强化农业基础支撑。启动蚌埠国家农业科技园区龙亢核心区“一城五园”建设，加快构筑区域农业科技创新聚集区、四化同步先行试验区、循环农业示范区。推进农田水利建设，创新农田水利管护体制机制，改造中低产田27万亩，新增有效灌溉面积8万亩。强化农业科技支撑，积极推行以专业化育秧、病虫害专业化防治等为重点的保姆式农业服务。加强与北京农学院等院校的战略合作，实施更多科研项目，培育更多有文化、懂技术、会经营的新型职业农民。鼓励发展农机专业合作社，新增农机总动力5万千瓦，主要作物综合机械化水平达70%以上。 
    聚力发展现代农业。深入实施粮食生产三大行动，调动和保护好农民种粮积极性，力争粮食生产再获丰收。在稳定粮食面积和产量水平的基础上，引导农民调整种养结构，发展设施农业，增加收入。新增标准化蔬菜园15个，规模养殖比重提高3个百分点。积极转变粮油加工业发展方式，着力推进主食产业化科学发展。坚持育龙头、拓市场、建基地、联农户“四位一体”，大力发展以食品加工为主的农产品加工业，加快固镇亿只肉鸡和皖北最大现代化肉牛、肉羊养殖产业化等项目建设，规划建设土特产品加工产业园，力争规模以上农产品加工企业突破300家。新增农民专业合作社100家、家庭农场100个。创建国家级现代农业示范区1个、省级农业产业化示范区1个。大力发展生态循环农业，着力节水、节肥、节药、节地、节能，无公害、绿色、有机农产品种植面积占比达50%以上。吸引社会资本投入乡村旅游和休闲农业，壮大农村集体经济。鼓励工商资本参与土地流转，引导土地向龙头企业、家庭农场等新型经营主体流转，提升规模化、专业化经营水平。新增土地流转20万亩。 
    深化农村综合改革。坚持和完善农村基本经营制度，依法保障农民对承包地占有、使用、收益、流转及承包经营权抵押、担保权利。不断探索农村土地集体所有制的有效实现形式，加快构建现代农业经营体系。开展农民住房财产权抵押、担保、转让试点。创新农村公共基础设施建设和管理机制，推动城乡要素平等交换和公共资源均衡配置。加快城镇户籍制度改革，有序推进农业转移人口市民化，让广大农民平等参与现代化进程，共同分享现代化成果。 
    (三)繁荣振兴服务业，重塑区域商贸重镇。 
    着力培育大市场、构筑大物流、提升大商贸、发展大旅游，推动服务业扩规模、上水平、添活力、增效益，巩固提升皖北商贸中心城市地位，社会消费品零售总额向500亿元大关冲刺。 
    加快区域性商贸中心建设。坚持一手抓高端商贸集聚辐射，一手抓便民商业体系完善，增强商贸服务整体功能。建成运营万达二期，改造提升光彩大市场，开工建设鹏欣水游城、新凤凰商城，打造东海大道高端商贸集聚带。加快实施天洋国际大厦、汇金中央水城、二马路及周边区域升级改造等项目，提升淮河路商圈。引进新企业，集聚新业态，激活升级淮河文化广场商圈。稳步推进小商品批发市场划行归市工作，促进义乌国际商贸城、海吉星等市场集聚发展。启动玉器市场划行归市。建成乡镇商贸中心11个、中心村连锁直营店25个。发展社区便民商业，完善社区商业服务功能，分区域建设特色风味美食街，打造一刻钟便民生活服务圈。新增限额以上商贸企业80户、服务业企业30户。 
    大力发展现代服务业。深入推进水运振兴工程，加快建设淮河新港、力源码头等物流集聚区，开工建设长淮卫临港产业园。着力引进中远、中集等一批知名物流企业，加快实施皖北徽商物流港、中储粮蚌埠直属库搬迁等项目。建设蚌埠铁路无水港，开通淮河水运集装箱班线。推进景区景点建设，支持现代花卉科技产业园创建国家4A级景区，加快实施花鼓灯嘉年华二期等项目，建成开放湖上升明月古民居博览园、大明文化产业园部分景点，旅游总收入突破100亿元。建设电商产业园，争取国美在线签约落户，推动传统企业商务电子化。创建省级服务外包示范区。加快建设4G网络，打造智慧城市。引导发展科技研发、工业设计等新兴行业，鼓励发展咨询、会展、快递、总部经济等现代服务业，大力培育养老、休闲、健康等消费增长点。 
    扩大对外交流合作。深化与长三角区域合作，主动对接上海自贸区，推动皖北四化协调发展先行区建设。全面实施大通关，完善外贸促进政策，加快蚌埠(皖北)保税物流中心建设，建成公共保税仓和出口监管仓，提高口岸对外开放能力。继续支持丰原集团等企业走出去发展。 
    (四)强化自主创新，支撑引领转型发展。坚持创新驱动，用足用活合芜蚌自主创新综合试验区大品牌、大载体、大平台，加快把科技优势转化为竞争优势，实现产业和科技两促进、双提升，赢得未来发展主动权。 
    突出企业创新主体地位。引导和支持创新要素向企业集聚，新增高新技术企业30户，高新技术产业产值达680亿元，战略性新兴产业产值突破500亿元。支持企业与大院大所合作，鼓励驻蚌单位和科技人员就地转化技术专利成果。新增院士、首席专家工作站5个。全面实施知识产权贯标工作。发明专利授权量420件，增长10%。 
    完善科技创新平台。加快推进浮法玻璃新技术等3个国家级重点实验室建设，争创国家信息消费产业发展研究院，新增省级以上研发平台6个，逐步实现高新技术企业研发机构全覆盖。发挥蚌埠电子信息产业技术研究院、硅基新材料产业技术研究院等平台作用，攻克MEMS加速度传感器、高性能消影玻璃等一批核心关键技术，促进产业链与创新链融合发展。扶持生产力促进中心、技术转移中心和专业咨询机构建设，提高科技公共服务能力。 
    强化人才工作。加大企业股权和分红激励试点政策实施力度，发挥人才金港优势，引进和培养符合产业发展方向的科技领军人才、实用人才，培育省“115”产业创新团队3支。继续深化与清华大学、复旦大学等高校全面合作，加快推进与安徽科技学院共建协同创新与人才培养基地。引导企业与院校共建学科专业，鼓励高校毕业生在蚌就业。 
    (五)坚持全域蚌埠，统筹推进新型城镇化。 
    把以人为核心的新型城镇化建设作为转型发展的重要引擎，注重新城拓展和老城改造同步推进、同样出彩，实施城市大建设项目180个、投资200亿元，着力构建以市区为核心、县城和园区为节点、重点镇和美好乡村统筹发展的城乡一体化格局。 
    拓展城市发展空间。放大区划调整效应，开工建设国道206马城段和省道306沫河口段，加快长淮卫、马城新区基础设施建设，增强市域综合承载力。推进高铁东站房、东广场设计建设，建成运营综合客运站，提升高铁新区综合服务功能。完善城南新区配套基础设施建设，实施朝阳南路二期、中环线城南段项目。加快建设长淮卫淮河大桥，力争开工建设延安路通河隧道，推进中环线与乳泉大道连接。 
    优化老城区功能。完成市区征迁400万平方米，交付净地2.5万亩。提升改造治淮路西段、国强路南段等老街，打通体育路东段、马场湖路等断头路，逐步完善市区路网格局。认真总结完善上一轮棚改工作好经验、好做法，按照精准核实面积、精细工作方案、精明控制成本、精美改造效果的要求，争取国开行和更多社会资本支持，启动实施新一轮棚改规划，推进大禹家园等12个保障性住房、市东新村等25个棚户区、金圩等15个城中村改造项目，开工建设各类保障性安居工程222万平方米，竣工190万平方米。加快老街区建筑外立面、公共配套设施改造，注入新的商业业态，让老城区传承历史记忆，焕发时代生机。 
    做大做美三县县城。继续推进重点设施向县域延伸，加快建设国道104五河段、省道307怀远段一级公路改造提升工程，谋划实施连接三县的东西中轴线快速通道。积极推进市县规划衔接，提升县城规划编制和实施水平。按照新区拉框架、老区增功能、整体上品位的思路，立足各自区位特点、资源禀赋、产业优势，统筹推进三县县城建设。怀远着力打造皖北最美县城，五河建设水韵独具皖北水乡，固镇以生态宜居的魅力新城迎接建县50周年。支持三县特色经济强镇发展，怀远重点加快龙亢产业开发区建设，谋划推进鲍集镇等中心镇发展；五河重点加快推进城关镇与头铺镇对接发展，谋划培育北部和东部中心镇；固镇重点加快南部中心镇和开发区建设，实现产业和城镇融合发展。 
    提高城乡管理水平。努力建成数字城管，全面实施网格化管理。推进市容保洁、市政设施维护市场化。推行生活垃圾桶装车载、渣土废料密闭运送，实现干道清扫保洁全程机械化，做到垃圾不见天、运输无抛洒。取缔三县县城客运三轮车。加强物业公司监管，提高物业管理水平。探索新型农村社区管理模式，开展乡村物业管理试点工作。实施老旧小区提升改造工程，按照实用、实际、实效原则，改造提升人行道，统筹推进道路整修、绿化亮化、停车设施等建设。启动智慧交通系统建设，推动道路交通管理集成化、信息化、智能化。 
    (六)狠抓项目投资，持续增强发展后劲。按照经济工作项目化、项目工作责任化要求，始终把项目作为加快发展的核心抓手，切实把各种资源、工作精力聚焦到项目上来，以项目提质提效推动经济社会更好更快发展。 
    加快重点项目建设。围绕前期项目抓推进，新建项目抓开工，续建项目抓进度，建成项目抓达效，分层分类推进项目建设，固定资产投资1000亿元以上。建成投产盛世光电玻璃产业园、和鼎轮胎一期等项目，加速实施冠宜国际箱包制造基地、联盟国际家博城、中纺油脂产业园等项目，开工建设国电蚌埠电厂二期、中华(固镇)食品工业园等项目。增强项目谋划的科学性、系统性、针对性，新增储备项目规模2000亿元。 
    加大招商引资力度。出台激励政策，推动企业围绕上下游、左右手以商招商、以企招商，形成引进一个、建好一个、带来一批的联动效应。围绕产业链缺失环节、短板之处以及整机、整器、整车类产业链末端、价值链终端产品，大力开展产业链招商，不断补链、延链、强链。针对高铁沿线、台湾等重点区域推动板块招商，探索合作设立产业园区新模式，整体承接产业转移。推动专业招商，发动全民招商，力争引进日丰电缆、万顺导电膜和山东方圆集团铜冶炼加工一体化等项目。全年实际利用省外亿元以上项目资金630亿元、外商直接投资11亿美元，每个县区至少引进30亿元以上项目1个。开展效能评级、效益评估、效果评价“三效三评”工作，提高招商引资质量。建立健全后续服务和预响应机制，突出重服务、讲环境的软性招商，让落户企业真心、舒心地参与蚌埠发展。 
不断拓宽融资渠道。引导金融机构继续扩大信贷规模，新增贷款180亿元，贷款余额超千亿。谋划建设皖北金融集聚区，启动建设科技金融创新园，引进1家股份制商业银行，新设2家融资性担保公司、证券公司营业部，全面完成县域农村合作金融机构改制工作。加大直接融资力度，支持各类债券发行和企业上市工作。拓宽中小企业商标质押贷款、动产抵押、股权出质、债转股等融资渠道。做大做强城投、蚌投、城开、交投、水投等融资平台，加强政府性债务管理，强化风险监测评估排查，严守不发生系统性金融风险的底线，把握结构、节奏和节点，科学、合规、高效融资。 
    放手发展民营经济。坚持把民营经济作为经济发展的重要增长点和支撑力量，认真落实权利平等、机会平等、规则平等的改革要求，进一步放宽市场准入，鼓励非公有制经济参与国有企业重组改造，支持非公有资本进入金融服务、公用事业、基础设施等领域。制定更加有力的扶持政策，最大限度地放开创业领域、放低创业门槛、放活创业方式，掀起新一轮全民创业热潮。新增私营企业1200户、个体工商户8000户，民营经济占GDP比重提高1个百分点。 
    (七)弘扬先进文化，打造特色文化名城。十分珍惜、悉心呵护蚌埠绵延7300年的文化根脉，充分挖掘和宣传大禹等具有蚌埠印记、蚌埠符号、蚌埠特质的优秀文化，彰显独具特色的文化魅力，铸造城市发展最根本、最持久、最难替代的核心竞争力。 
    加强思想道德建设。高举中国梦精神旗帜，扎实推进社会主义核心价值体系建设。以蚌埠历史文化为基、时代精神为魂，提升市民价值观念、思维方式、伦理情趣，弘扬以创新进取、责任担当、务实诚信、合作共赢为核心的先进思想文化，让更多的蚌埠人成为文化的守望者、传承者、创造者。深化文明城市创建和乡风文明提升，搞好文明单位、文明村镇、文明社区、文明家庭、道德模范评选，广泛开展志愿者服务活动，打造区域性文明示范城市。发挥主流媒体阵地作用，加强对社会热点的舆论引导，弘扬主旋律，传播正能量。 
    繁荣发展文化事业。引入竞争机制，鼓励社会力量和社会资本参与公共文化服务体系建设，促进基本公共文化服务标准化、均等化。加强传统文化研究、保护和利用，扩大双墩、大禹等淮河文明特色文化影响力。申办第五届中国(蚌埠)花鼓灯歌舞节暨国际民间歌舞艺术交流周和中国(蚌埠)玉文化节。开放新博物馆、档案馆、规划馆和市民广场，进一步规划科技馆、文化馆、体育中心，分区域启动建设百姓健身场馆。整合乡镇文化站、电子阅览室、农家书屋等农村公共文化设施，试点建设一批乡村文化乐园。继续开展“温馨蚌埠、欢乐珠城”系列演出，组织千场文艺千村行活动，持续活跃群众竞技场、百姓大舞台。 
    培育壮大文化产业。深化文化体制改革，加快国有经营性文化单位公司制、股份制改造，鼓励民营文化企业发展，建立多层次文化产品和要素市场。整合、集聚和再造西部城区丰富的历史人文资源，突出打造荆涂淮河湾，做大、做实、做美大禹文化产业园。规划和建设双墩、禹墟、垓下等遗址公园，推进冯嘴子村等文化旅游生态村建设，着力打造一批叫得响、立得住、传得广的特色文化品牌。加快实施星宇文化创意产业园二期等项目，大力发展动漫、数字等朝阳产业，挖掘、再现深厚文化底蕴，弘扬、传播城市特色文化。 
    (八)推进生态靓市，营造美丽生态家园。坚持从五位一体高度，突出抓好生态文明建设，争创国家生态文明先行示范区，建设水在城中、城在林中、城依山、水映城的山水园林城市，打造外地人羡慕、投资者向往、本地人自豪的美丽生态家园。 
    大力活水。实行最严格水资源管理，落实水资源开发利用控制、用水效率控制、水功能区限制纳污三条红线。加强河湖管理，推进淮河、天河湖、张公湖、龙子湖“一河三湖”为主的城市水环境整治。谋划开通天河-龙子湖、天河-张公湖环城水系，规划建设天龙河，启动实施天公河工程，让灵动之水跃动魅力珠城。加快三汊河湿地公园保护性开发建设，加大芡河、四方湖、沱湖等水质保护和水岸基础设施建设力度，让清澈水源滋养百姓身心。大力实施退网还湖工程，推进现代化、智能型养殖基地建设。落实渔民上岸后续清岸、美岸工程，加大城区内淮河两岸滩涂环境综合整治力度。 
    全力增绿。认真落实千万亩森林增长工程，继续实施绿满珠城行动，开展“村村绿”和绿色家园示范建设活动。成片造林15万亩，建设森林长廊80公里，新建农田林网31万亩，森林覆盖率达16.1%以上。加快陶山植物园建设，高标准建设淮河北岸滨河绿地景观带和黄山大道、淮上大道两侧绿道。结合棚户区和旧城改造，实施拆墙透绿、拆违补绿、退地还绿等增绿工程。推进“三线三边”环境整治，新启动20个美好乡村中心村建设。 
    强力减污。开展节能减排全民行动，倡导文明、节约、绿色、低碳生活方式和消费模式。完成一污厂、三污厂和怀远、固镇污水处理厂提标改造工程，启动蚌埠铜陵产业园、天河西片区污水处理厂建设。完善农村排污管网及配套处理设施，扶持和引导农民建设生活污水净化沼气池。推行餐厨废弃物无害化、资源化处置。开展新建住宅小区集中供暖试点。强力落实大气污染防治行动计划，坚决实行全年全市全面秸秆禁烧，建立与凤阳等周边地区大气污染联防联控协作和雾霾天气应急机制，让城乡居民仰望蓝天、拥抱绿色、感受清新。 
    (九)着力改善民生，不断提高群众幸福感。 
    注重发展成果与人民共享，以民生改善检验发展质量，坚持制度安排与项目建设并抓、托底与抬高并举，加大民生投入，让百姓不断感受到、享受到大发展、大建设给生产、生活带来的新变化。 
    深入实施民生工程。继续实施33项省级民生工程，注重抓好就业促进工程、城乡居民大病保险等4个新增项目。 
    各位代表，我们将继续按照以人为本、民生为先的要求，始终把百姓装在心里，把心放在百姓家里，从群众最关心、最需要、最实惠的事情做起，实施好12项市级民生工程。新增、更替城乡环保公交车175辆，提升改造公交站台200个，对7类特殊群体实行乘车免费优惠政策。新建、改造路灯8000盏、城市公厕100所。实施环境保护能力建设工程。新建、改造标准化农贸市场4个，完成20家农产品进超市、社区便利店工作，新建农副产品平价直销店26个。实行城乡居民临时救助。对社会办养老服务机构给予每个床位建设一次性补助1000元。继续支持示范社区创建，奖补新建设施蔬菜基地，资助贫困大学生、困难家庭学龄前儿童入园，对城乡80岁、百岁以上老人给予补贴，实施一户多残家庭生活补助。 
    协调发展社会事业。推进素质教育，完善教育集团化办学体制。北师大蚌埠附属学校开学招生。加快蚌埠职教园建设，完成市特教中心新校区搬迁，开工建设职教中心新校、蚌埠汽车工程学校，恢复建设卫生类中职学校。做好高校教学科研和学生生活场所安装空调设备工作。实施医疗卫生提升工程，着手启动市级公立医院综合改革，建成市中医院新院区和皖北食品药品检验中心，开工建设上海长征医院合作医院、蚌医二附院新院区、一院综合病房大楼，推进与复旦大学合作建设妇女儿童临床中心和附属儿童医院蚌埠分院。加大妇女儿童“两纲”实施力度，规划建设新儿童公园。严格落实生育政策调整工作。实施青年创业就业“1248”工程，实现基层老年协会全覆盖，推进残疾人社会保障和服务体系建设。规划和启动建设公益性公墓。广泛开展全民健身行动，让群众成为体育活动的实践者、受益者。积极备战、认真参赛，力争在省第十三届运动会上取得好成绩。扎实做好第三次经济普查工作。加快“金审”三期工程建设。推进住房公积金征缴扩面。开展民族团结进步示范社区创建活动。提升外事、侨务、港澳台事务综合服务水平。重视地方志编纂工作，充分发挥存史资政、教化风气作用。在全省率先建成数字档案馆。深入开展拥军优属、拥政爱民活动，不断加强国防动员和后备力量建设。加快气象现代化建设，发挥气象综合效益。大力推进人防建设，加强防震减灾工作，完善以应急平台为重点的应急指挥体系。认真开展危化、道路交通、建筑施工等安全生产专项治理，强化食品药品安全监管，有效遏制重特大事故发生。 
完善社会保障体系。深入推进城乡居民收入倍增规划，千方百计增加城乡居民尤其是中低收入者收入。健全促进就业创业体制机制，全面组织实施孔雀计划，以创业带就业，以就读促就业，全年新增城镇就业6万人。建立更加公平可持续的社会保障制度，推动医疗保险门诊统筹和付费方式改革，积极落实大病救助、五保供养等政策，推进城乡居民养老保险、最低生活保障等提标扩面，低保标准和覆盖面达到全省平均水平。 
    不断创新社会治理。改进社会治理方式，全面加强系统治理、依法治理、综合治理、源头治理。完善基层社会管理服务，深化社区行政管理体制改革，开展社区基本公共服务全覆盖试点。创新有效预防和化解社会矛盾体制，改革信访工作制度，及时就地解决群众合理诉求。推进平安蚌埠建设，完成主要街区监控设施全覆盖，依法严密防范和惩治各类违法犯罪活动。 
    三、改革创新，全面提升政府工作水平 
    各位代表，民之所望，施政所向。站在新起点，面对新期待，我们将朝夕勤勉，永不懈怠，勇为改革担风险，敢为发展尽责任，善为百姓谋福祉，努力打造人民满意政府。 
    (一)凝神聚气、开拓进取，增添赶超跨越新动力。时刻保持抢抓机遇的紧迫感和知难而进的精气神，以更大的勇气和智慧冲破思想观念的障碍、突破利益固化的藩篱，寻求改革共识最大公约数，汇聚加快发展强大正能量。坚定重返全省第一方阵的信心和决心，向先进地区看齐，逐项对标追赶，逐层传递压力，各项工作创一流，条条战线争上游。坚持问题导向，建立问题“销号”制度，用问题的化解推动事业的前进，靠责任的压实成就发展的愿景。不断提升开放意识和开发能力，在更高层面、更广领域、更大空间谋划蚌埠、创意蚌埠、发展蚌埠。放大“厚德蚌埠、好人之城”效应，激发群众内心深处的美好情感和道德意识，让城乡文明与经济发展同频共振，让珠城百姓与蚌埠崛起相融共生，充分展示全体蚌埠人重振雄风、再创辉煌的大气、勇气和底气。 
    (二)突破自我、改革创新，激发区域竞争新优势。坚持“胆子要大、步子要稳”的战略战术，推进新一轮政府职能转变和机构改革，积极谋划、快速推进看得准、很急需、市级层面能定的改革，紧密衔接、迅速落实省委、省政府确定的各项改革。大刀阔斧地削减行政审批，全力推进行政审批标准化和流程再造，为企业松绑，向市场放权，让群众方便。完成市县工商、质监行政管理体制调整和食品药品监管体制改革。加快推进事业单位分类改革。推行大部门制和市直部门大科制改革，集中主要职责、集成相关职能、集约人力财力。探索“负面清单”管理模式，实行政府及其工作部门权力清单制度。开展全市行政事业单位及所投资企业资产大清查，千方百计盘活国有存量资产。推行全程公开合理低价招投标模式，全面实行标后监管第三方评估机制，保障盖好房、铺好路、带好人。加大政府购买服务力度，公平对待社会力量提供公共服务。大力培育发展社会组织，推动行业协会商会和中介服务组织与行政机关真正脱钩。紧扣市场在资源配置中的决定性作用，加强市场监管，推进公平准入，维护公平竞争，激发市场主体活力。 
    (三)转变作风、提升效能，展现干事创业新面貌。严格执行中央八项规定，厉行勤俭节约，反对特权思想，严控“三公”经费支出，切实减少会议、文件和事务性活动，把更多的财力和精力留给基层、留给群众、留给项目。扎实开展群众路线教育实践活动，集中解决形式主义、官僚主义、享乐主义和奢靡之风“四风”问题，推动改进工作作风、密切联系群众制度化常态化。加强机关效能建设，强化“我负责、我来办、马上办”的理念，坚持立说立行、雷厉风行，杜绝不作为、摒弃慢作为，用干部的效率指数换取群众的幸福指数。深入实施服务企业、服务百姓“百千万”行动，推进科技、金融、文化、交通等服务进百企、帮千家、助万户，让企业经营安心，让客商投资放心，让百姓生活舒心，让每一个蚌埠人对未来发展充满信心。 
    (四)依法行政、科学施政，树立法治政府新形象。推进依法治市，带头学法、尊法、守法、用法，增强运用法治思维和法治方式推动发展、化解矛盾能力。注重依法办事和依法维权相结合，深化行政执法体制改革，提高执法和服务水平。坚持依法科学民主决策，健全决策咨询制度，完善规范性文件、重大决策合法性审查机制，把公众参与、专家论证、风险评估和集体讨论决定作为必经程序。立足打基础、利长远，加强对重大问题的深层次、战略性和系统性研究，完善有利于科学发展的目标体系、考核办法、奖惩机制，有序、有效、有力推进各项重点工作，杜绝拍脑袋、拍胸脯。依法扩大政务公开和信息公开，做优市长热线服务中心、政风行风热线等平台，开门听民意、问民需、解民忧，增强政府公信力，提升群众信任度。 
    (五)接受监督、廉洁自律，开创携手并进新局面。坚决维护组织的权威和市委的领导，自觉接受人大法律监督和工作监督、政协民主监督、新闻舆论监督、网络舆情监督、社会公众监督，高度重视司法监督，巩固和发展良好的政治生态。坚持重大事项、重大决策及时向人大及其常委会报告、与政协协商，虚心听取各民主党派、工商联、无党派人士及各社会团体的意见。认真贯彻执行人大及其常委会决议决定，积极办好人大代表议案、建议和政协委员提案，主动邀请人大代表、政协委员对政府重点工作、重大项目以及群众关心的热点难点问题，开展调研、视察、询问和专题协商，合力推动各项工作落实。全面落实党风廉政建设责任制，深入推进廉政风险防控管理，强化行政监察和审计监督，完善公共资源交易管理、财政性建设资金使用等制度，把权力关进制度笼子，形成不敢腐的惩戒机制、不能腐的防范机制、不易腐的保障机制。 
    各位代表，我们深知，前进的道路充满艰辛，发展的责任重如泰山。我们深信，任何困难也动摇不了赶超跨越的坚定决心，任何挑战也阻挡不了加速崛起的坚实步伐。让我们在中共蚌埠市委的坚强领导下，振奋精神，开拓进取，扎实苦干，继续朝着重返全省第一方阵的目标阔步向前！
